# Transition from Acute to Chronic Tinnitus: Predictors for the Development of Chronic Distressing Tinnitus

**DOI:** 10.3389/fneur.2017.00605

**Published:** 2017-11-20

**Authors:** Elisabeth Wallhäusser-Franke, Roberto D’Amelio, Anna Glauner, Wolfgang Delb, Jérôme J. Servais, Karl Hörmann, Ines Repik

**Affiliations:** ^1^Otorhinolaryngology, Phoniatrics and Audiology, Medical Faculty Mannheim, Heidelberg University, Mannheim, Germany; ^2^Department of Internal Medicine IV and Neurocenter, Saarland University Medical Center, Saarland University, Homburg, Germany; ^3^Otorhinolaryngology, University Medical Centre Mannheim, Mannheim, Germany

**Keywords:** recent-onset tinnitus, prospective study, acute-chronic transition, hearing impairment, depression, anxiety, coping with illness

## Abstract

**Background:**

Acute tinnitus and its transition to chronic tinnitus are poorly investigated, and factors associated with amelioration *versus* exacerbation are largely unknown. Aims of this study were to identify early predictors for the future development of tinnitus severity.

**Method:**

Patients with tinnitus of no longer than 4 weeks presenting at an otolaryngologist filled out questionnaires at inclusion (T1), as well as 3 (T3), and 6 months (T4) after tinnitus onset. 6 weeks after onset, an interview was conducted over the phone (T2). An audiogram was taken at T1, perceived tinnitus loudness, and tinnitus-related distress were assessed separately and repeatedly together with oversensitivity to external sounds and the levels of depression and anxiety. Furthermore, coping strategies with illness were recorded.

**Results:**

Complete remission until T4 was observed in 11% of the 47 participants, while voiced complaints at onset were stable in the majority. In the subgroup with a relevant level of depression at T1, tinnitus-related distress worsened in 30% until T4. For unilateral tinnitus, perceived loudness in the chronic condition correlated strongly with hearing loss at 2 kHz on the tinnitus ear, while a similar correlation was not found for tinnitus located to both ears or within the head.

**Conclusion:**

Results suggest early manifestation of tinnitus complaints, and stress the importance of screening all patients presenting with acute tinnitus for levels of depression and tinnitus-related distress. Furthermore, hearing levels should be monitored, and use of hearing aids should be considered to reduce tinnitus loudness after having ascertained that sound sensitivity is within normal range.

## Introduction

Subjective tinnitus is an acoustic perception which is not caused by an external sound source, but by aberrant activation within the auditory system (1). This type of tinnitus is rather common, and because of its subjective nature, characteristics of the tinnitus are mostly derived from patients’ reports. Tinnitus is usually associated with hearing loss (HL) as detected by pure tone audiometry, but the perceived severity bears only weak to moderate relations with hearing thresholds and other psycho-acoustically determined features of the tinnitus, while high tinnitus-related distress is often associated with poor mental well-being (2–4). As tinnitus may severely impair life quality of affected individuals, it is crucial to identify factors that are predictive for the development of disabling tinnitus before it becomes a chronic condition.

There exists a multitude of cross-sectional studies on factors associated with chronic tinnitus (e.g., this Frontiers Topic), whereas only few studies have attempted to assess participants with acute tinnitus (5–7) including a study that investigated tinnitus patients one year after their first visit to a clinic if they entered the clinic within 6 months of tinnitus onset (8). Therefore, time course as well as mechanisms involved in the transition from the acute to the chronic condition are unknown, and factors predisposing for the development of a chronic, disabling, or decompensated tinnitus are mostly inferred from retrospective reports. Furthermore, studies on acute tinnitus usually were concerned with tinnitus following sudden HL (6, 7) or acute acoustic trauma (9), which appear to be distinct as indicated by high remission rates which may reach 70% (10). High remission rates concomitant with an incidence of HL are likely related to partial recovery of hearing function in the first weeks after a temporary threshold shift (11), but not all patients can relate tinnitus onset to worsening of their hearing. Therefore, remission rates may be lower and it may not be advantageous to wait for spontaneous remission but take action early on to prevent the development of a chronic disabling tinnitus.

Across many cross-sectional studies on chronic tinnitus, psychiatric comorbidity in particular depression is the most frequent condition for those with severe tinnitus (3, 4, 12, 13). Therefore, it has been suggested that pre-existent psychiatric comorbidity may foster decompensation (5), a notion that is corroborated by the few studies with early interventions, which suggest that psychological interventions may prevent the development of disabling tinnitus to some extent (5, 14–16). In contrast, tinnitus related to acute worsening of hearing thresholds may be susceptible to pharmacological interventions (17, 18). However, effectiveness of all these interventions is challenged because of unknown remission rates.

We set out to investigate the development of acute tinnitus within the 6 months after its first appearance, i.e., during transition from the acute to the chronic condition (19). As tinnitus is a phenomenon associated with the ears, patients experiencing it for the first time are likely to seek help by an otolaryngologist, at least in a country where the general health system allows them to do so without extra cost. Therefore, we aimed to investigate the history of tinnitus in patients seen by otolaryngologists within 4 weeks after tinnitus onset. Concerned with these patients the otolaryngologist has to decide on the type of treatment that is suitable for the particular patient knowing that some may lose their tinnitus and that most will not bother with it in the long run, while a low percentage will develop a distressing tinnitus that severely affects their quality of life. Because early interventions may prevent the acute symptoms to become a chronic disabling condition (5, 14–18), it is crucial to find out early which patients are at risk to develop a disabling chronic tinnitus, and which interventions may be suitable for the individual patient. To our knowledge, the present study represents the first attempt to assess in detail the characteristics of this tinnitus population including spontaneous recovery.

Factors to be investigated in relation to tinnitus were derived from the literature on chronic tinnitus and from our experience with acute tinnitus patients (5, 14). Particular focus on the instruments used was their applicability by otolaryngologists. In addition to pure tone audiometry, self-report measures on subjectively perceived tinnitus loudness and tinnitus-related distress were used in conjunction with screening instruments for the levels of depression and anxiety and a questionnaire on coping with illness.

Aims of the study are to

(1) describe characteristics of acute tinnitus patients that seek medical help from otolaryngologists.
(2) describe the time course of transition from the acute to the chronic condition.
(3) find out whether a high level of depression at tinnitus onset promotes development of a decompensated chronic tinnitus, and whether individuals at risk can be identified early on.
(4) identify maladaptive ways of coping with tinnitus,
(5) describe factors associated with spontaneous remission.

Preliminary data based on 28 participants have been published already (20).

## Materials and Methods

### Procedure

Between 6/2013 and 4/2016, all first-time presenters with tinnitus at the Ear, Nose and Throat Clinic of the University Medical Centre Mannheim and at 4 participating ENT-practices in the region (see acknowledgements) were asked if they were willing to participate in the study. All willing to participate and meeting inclusion criteria were informed about aims and time required for the study. Inclusion criteria were first time tinnitus of no longer than 4 weeks, age above 18, and sufficient command of German. Exclusion criteria were neurological diseases and simultaneous psychological therapies. Participants were informed that in case they were developing distressing tinnitus, they would be assisted to contact a specialized outpatient care for tinnitus patients at the Central Institute of Mental Health, Mannheim. After completion of the study, those who wished a feedback were informed about their level of tinnitus-related distress. Study design and procedures were approved by the Ethics Committee II of Heidelberg University at the Medical Faculty Mannheim.

After giving written informed consent, the audiogram that was routinely taken was archived with the study documents. In addition, study participants filled out a comprehensive paper and pencil questionnaire with the instruments outlined below, and they gave the exact date when they first noticed their tinnitus (T1). Questionnaires were collected at a regular basis by the authors. A telephone interview was conducted 6 weeks after the recorded date of tinnitus onset (T2). During this assessment participants could ask tinnitus-related questions. Hence, this interview can be seen as additional counseling which was received by each of the participants. At T2, the interviewer remembered each participant that two more questionnaires were going to be sent within 3 (T3) and 6 (T4) months after tinnitus onset. Questionnaires were accompanied by pre-stamped return envelopes. Patients who had not answered within 2 weeks after sending these questionnaires were contacted by phone, and asked to return the filled-in questionnaire.

### Instruments

At T1, demographic information was collected, and participants were asked about date and circumstances of tinnitus onset. At all assessments, they answered questions regarding tinnitus laterality and character, aggravation, or alleviation in relation to first appearance and previous treatments. In addition, the self-report measures described below were administered.

### Audiometry

At T1, a pure tone audiogram was taken separately for each ear according to general procedures in otolaryngology practices in Germany for the standard frequencies between 0.25 and 8 kHz. When no response to stimulation was evoked, the measurement was considered to be impossible, and for calculation, a value of 100 dB was used.

### Outcome-Variables on Tinnitus Severity

Since clinically there appear to be significant differences between patients who focus on sensory aspects of the tinnitus and those who focus on functional disability and handicap (4, 21), we used separate measures for those two aspects. Tinnitus loudness as perceived by the participant (T-NRS) was recorded at T1, T3, and T4 on a Numeric Rating Scale [NRS (21)] with the anchors “heard only during silence” (0) and “louder than all other sounds” (10).

Tinnitus-related distress was assessed with the 12-item Tinnitus Questionnaire [Mini-TQ12 (22)]. According to Zeman et al. (23), the Mini-TQ12 shows satisfying psychometric results and is sensitive to changes in tinnitus severity. For the Mini-TQ12, each of 12 items may be answered with “true,” “partially true,” and “not true,” scored as 2, 1, and 0, respectively. Tinnitus-related distress is the sum of points given to the 12 items and ranges from 0 (no distress) to 24 (maximal distress). According to Hiller and Goebel (22), 4 grades are discerned with grade 1 (0–7) representing no or mild distress and grade 2 (8–12) representing moderate distress. The range of 0–12 is also classified as compensated tinnitus, while decompensated tinnitus includes grades 3 (distressing tinnitus: 13–18), and 4 (severely distressing tinnitus: 19–24). In order to prevent a potential aggravation when asking for specific tinnitus-related consequences on life at T1, the Mini-TQ12 was used at T2, i.e., 6 weeks after tinnitus onset, for the first time and then again at T3 and T4. During the telephone interview at T2, the twelve Mini-TQ12 items were read to the participant together with the response options. Questions were repeated as needed.

As a second instrument to address tinnitus-related distress, we employed the Sheehan Disability Scale (SDS, 24) at T1, T3, and T4. In contrast to the Mini-TQ12, the SDS circumvents the potential risk of tinnitus aggravation. It assesses functional impairment in work/school (SDS1), social (SDS2), and family life (SDS3) on three 10 point visual analog scales that present numeric and verbal descriptive anchors in addition. There is no cutoff but scores of ≥5 on any of the scales; and high scores in general are associated with significant functional impairment. A SDS total sum score was not calculated, because not all participants worked and therefore not all reported a SDS1 score. The SDS was validated and shown to be sensitive to treatment-related changes in a variety of conditions (24–27) but has not been used in relation to tinnitus before.

### Outcome Predictors

Outcome predictors were chosen according to the literature on chronic tinnitus and according to our experience with acute and chronic tinnitus patients.

#### Depression and Anxiety

Depression and anxiety are common comorbidities of disabling tinnitus (3, 4). Levels were assessed at T1, T3, and T4, with the freely available PHQ9 scale for depression and the GAD7 scale for generalized anxiety disorder (GAD) [(28), German (29)]. Both scales have been validated in primary care populations [PHQ9 (30); GAD7 (31)], there exist normative data for Germany (29, 32), and the scales have been used in tinnitus studies (4, 33). GAD is the most frequent anxiety disorder in primary care, and the GAD7 is sensitive also to other common forms of anxiety (29). Subjects were asked how often, during the past 2 weeks, they have been bothered, and response options for both scales were “not at all,” “several days,” “more than half the days,” “nearly every day” scored as 0, 1, 2, and 3, respectively. PHQ9 scores range from 0 to 24, and GAD7 scores range from 0 to 21. In both scales, lower score points indicate favorable conditions, while sum scores ≥10 indicate potential problems in these fields, and scores of 15 or above in the PHQ9 scale are classified as Major Depression.

#### Coping with Illness

The way people cope with their tinnitus was shown to be related to adjustment to the tinnitus (34), and it may be improved by psychological interventions (5, 14, 35). Coping strategies with illness were assessed with the German-language Freiburg Questionnaire of Coping with Illness (FKV: 36) at T1, T3, and T4. This 35-item questionnaire covers the following coping strategies in 5 subscales: depressive coping, active problem-oriented coping, distraction and self-affirmation, religiousness and search for meaning, and trivialization and wishful thinking. Answers are scored on 5-point Likert scales with higher scores indicating a higher intensity of coping in a particular domain. Mean scores in subscales are used for analysis. The questionnaire has good internal consistency with a Cronbach’s α of 0.68 and 0.77 for single scales (36) and has been used in relation to tinnitus (37).

#### Oversensitivity to External Sounds

As tinnitus is often associated with diminished sound-level tolerance or hyperacusis (38), oversensitivity to external sound was assessed on a 0–10 NRS (Hyper-NRS) with the extremes “normal” (0) and “extremely sensitive” (10) at T1, T3, and T4.

### Statistics

Descriptive statistics, and tests for statistical significance were performed with SPSS version 24 (SPSS/IBM, Chicago, IL, USA). Changes of the tinnitus variables, sound sensitivity, and mental health factors during the study were monitored, and differences were tested for statistical significance with the Global Linear Model for repeated measures and Bonferroni corrected *post Hoc* tests in the case of normal distribution, or with non-parametric Friedman and *post hoc* Wilcoxon tests if the variables were not normally distributed. A *p* value below 0.05 was considered to be statistical significant, and a *p* below 0.01 was considered as statistically highly significant. Bivariate correlations were performed between predictor variables at T1 and outcome variables at T4. Since this was an exploratory study, bivariate *p*-values of correlations are presented along with an indication which *p*-levels achieve the Bonferroni-corrected significance level. Because 54 bivariate correlations were performed, the Bonferroni-corrected *p*-value to reach statistical significance was 0.0009. Step-wise regression analyses were performed separately for each of the 5 outcome variables. Predictor variables with significant correlations, or correlations reaching a Pearson correlation coefficient above 0.300 and an uncorrected *p*-value below 0.05 with an outcome variable were included in the final step-wise regression analysis for this outcome.

### Participant Characteristics

Fifty-seven participants were included in the study, three were excluded because the time interval between tinnitus onset and T1 was longer than 4 weeks, and one participant was excluded for taking part in a psychotherapeutical tinnitus intervention in a tinnitus clinic during the study interval. Six participants dropped out, one obviously after his tinnitus was not present at T2 anymore and the others without notice, leaving 47 (82.5%) with complete data that contributed to the results. Their sociodemographic data are given in Table 1. Most lived in a long-term partnership, education standard was high and no one used a hearing aid.

**Table 1:** Sociodemographic factors at T1.

	Persisting tinnitus (*N* = 41)	Remitting (*N* = 5) or fluctuating (*N* = 1) tinnitus
Sex (m/f)	53.2%/46.8%	83.3%/16.7%
Age in years [mean (SD)]	41.4 (15.6)	41.0 (14.8)
Range	21–79	23–61
**Cohabitation**		
Single	8.5%
Cohabitating	91.5%	100%
**Highest educational achievement**		
None	2.1%	–
Basic	8.5%	33.3%
Middle	19.1%	–
University entrance	27.6%	33.3%
University degree	42.6%	33.3%
**Occupational activities**		
Working	74.5%	83.3%
Scholastic	10.6%	16.7%
Working in own household	6.4%	
Retired	6.4%	
Inability to work	4.3%	
Not working because of tinnitus	0%	
**Other chronic health conditions**		
No/yes/no statement	68.3%/29.3%/2.4%	66.7%/33.3%

## Results

Potential underlying causes of the tinnitus were unknown to most participants. Tinnitus onset was associated with a reduction of hearing ability that was noticeable to the patient in 24%. Some presented with vertigo which was not the major complaint, however, and no one was diagnosed with Meniere’s. Two participants had an ear infection. Of note is that most of the study participants did not have a very loud or very distressing tinnitus when seeking medical advice (Table 2).

**Table 2:** Comparison of subgroups with persisting and remitting tinnitus.

Assessment at T1/T2	Persisting tinnitus (*N* = 41)	Remitting tinnitus (*N* = 6)
T-NRS	3.95 ± 1.66	3.17 ± 1.72
Mini-TQ12	8.32 ± 5.76	No tinnitus
Sheehan Disability Scale (SDS)1	3.82 ± 2.43	2.00 ± 2.0
SDS2	3.38 ± 2.15	2.00 ± 1.5
SDS3	3.60 ± 2.23	2.00 ± 2.10
Hyper-NRS	4.66 ± 2.77	3.00 ± 1.50
PHQ9	7.24 ± 4.76	3.83 ± 1.60
GAD7	5.66 ± 3.85	3.00 ± 2.50
Audiogram mean right ear	17.74 ± 9.65	15.77 ± 4.25
Audiogram mean left ear	19.51 ± 9.44	14.13 ± 2.22

*Means and SDs are shown*.

### Tinnitus Therapies during the Study

Therapies were prescribed by the ENT-physician and were independent of study participation except for psychological/psychotherapeutic therapies that let to exclusion. Of the six participants with remitting tinnitus, one took an antibiotic for middle ear infections, another two received corticosteroid infusions, and one took medication enhancing blood flow, while two did not undergo a tinnitus-specific therapy.

Of the 41 participants with persisting tinnitus, 23 underwent a tinnitus-specific therapy until T2, and an additional 2 participants had received a tinnitus-specific therapy until T4. Fourteen received corticosteroids *via* infusions or orally, 9 took a Gingko preparation, 4 were prescribed Arlevert or Dusodril and one received an antibiotic for middle ear infections. Two underwent acupuncture and 2 received physical therapy. No one was prescribed a hearing aid. Nine participants used several therapies in combination. Tinnitus onset appeared suddenly more often in the group that underwent a tinnitus-specific therapy (84%) as compared to those without (56%). Averages of T-NRS, SDS1-3, PHQ9, and GAD7 scores at T1 were higher in the group that underwent a tinnitus-specific therapy. Group differences and most importantly group differences in the reductions of tinnitus complaints between T1 and T4 did not reach statistical significance, however. Therefore, data of both groups were pooled.

### Tinnitus Remission

**Figure 1:**
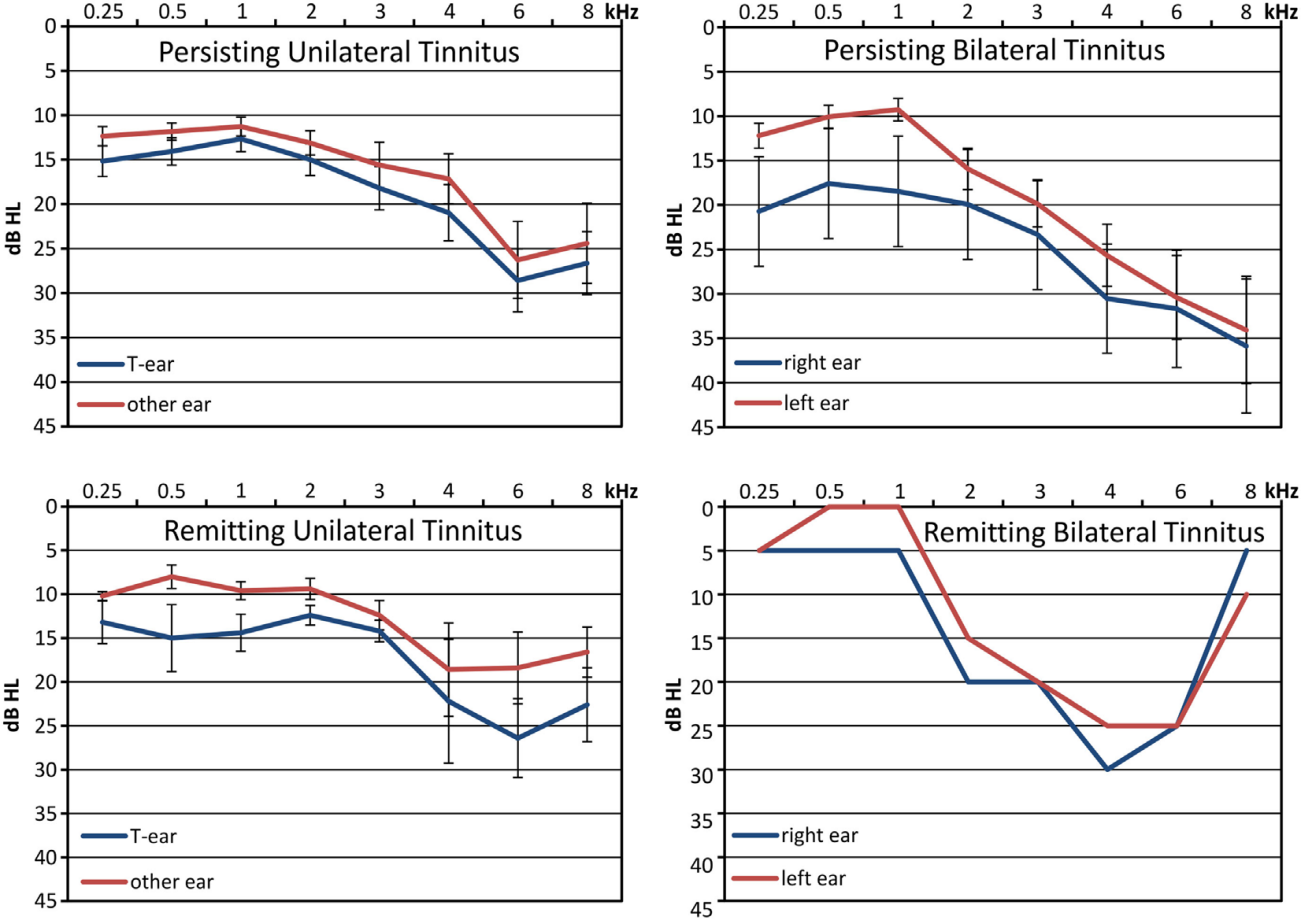
Pure Tone Audiograms for groups with persisting and remitting, unilateral and bilateral tinnitus: shown are means with respective standard errors. Unilateral tinnitus: tinnitus heard from one ear, bilateral tinnitus: tinnitus heard on both ears or within the head. An audiogram was not available from one individual with persisting unilateral tinnitus and from another one with persisting bilateral tinnitus.

Complete remission of the tinnitus until T4 occurred in 5 study participants, and tinnitus was absent at T2 and T3 in another one but returned until T4. One presented with a middle ear infection at T1 that was treated successfully with an antibiotic and the tinnitus had vanished until T2. Two reported sudden HL, they were treated with steroid infusions, their hearing recovered and the tinnitus was gone before T2. Reasons for remission in the others are unknown as well as for the fluctuation of tinnitus in another one for whom the tinnitus had reappeared at T4. Further characteristics of the group with tinnitus remission are shown in Tables 1–3 and in Figure 1. Although average age was similar to the group with persisting tinnitus, maximum age was lower, tinnitus onset was more often associated with a noticeable HL, and a higher percentage localized their tinnitus to one ear as opposed to both ears or within the head (Table 3). Whereas perceived tinnitus loudness was similar to the group with persisting tinnitus, tinnitus-related distress and oversensitivity to sound were lower (Table 2), coinciding with personal statements regarding changes in tinnitus loudness and distress between onset and T1. No one with remitting tinnitus reported an increase in tinnitus loudness or distress. Most notable were also the lower levels of depression and anxiety (Table 2).

**Table 3:** Comparison of tinnitus characteristics between groups with persisting and remitting tinnitus.

Tinnitus characteristics at T1	Persisting tinnitus (*N* = 41)	Remitting (*N* = 5) or fluctuating (*N* = 1) tinnitus
**Days between onset and T1**		
Mean ± SD	14.9 ± 8.5	7.7 ± 10.7
Range	1–28	2–29
Sudden onset	30 (73.2%)	3 (50%)
Subtle onset	11 (26.3%)	3 (50%)
**Onset**		
Associated with hearing loss (HL)	7 (17.1%)	3 (50%)
Not associated with HL	34 (82.9%)	3 (50%)
Permanent	28 (68.3%)	4 (66.7%)
Intermittent	13 (31.7%)	2 (33.3%)
**Localization**		
Right ear	10 (24.4%)	4 (66.7%)
Left ear	16 (39.0%)	1 (16.7%)
Both ears	10 (24.4%)	1 (16.7%)
Within head	5 (12.2%)	1 (16.7%)^a^
**Change in perceived loudness between onset and T1**		
None	28 (68.3%)	2 (33.3%)
Less loud	4 (9.8%)	4 (66.7%)
Louder	7 (17.1%)	0
No statement	2 (4.9%)
**Change in tinnitus-related distress between onset and T1**		
None	19 (46.3%)	2 (33.3%)
Less distressing	3 (7.3%)	1 (16.7%)
More distressing	11 (26.8%)
No statement	8 (19.5%)	3 (50%)

*Differences between these groups exist for localization of the tinnitus, a higher frequency of sudden onset, a higher percentage with no change or increasing loudness and distress between onset and T1*.

*^a^Same participant that experienced tinnitus on both ears*.

As data on tinnitus variables was not available for all time points for participants with remitting tinnitus, the following analyses on the progression on tinnitus loudness and distress was performed with the remaining 41 with persisting tinnitus until T4.

### Acute Condition

**Figure 2:**
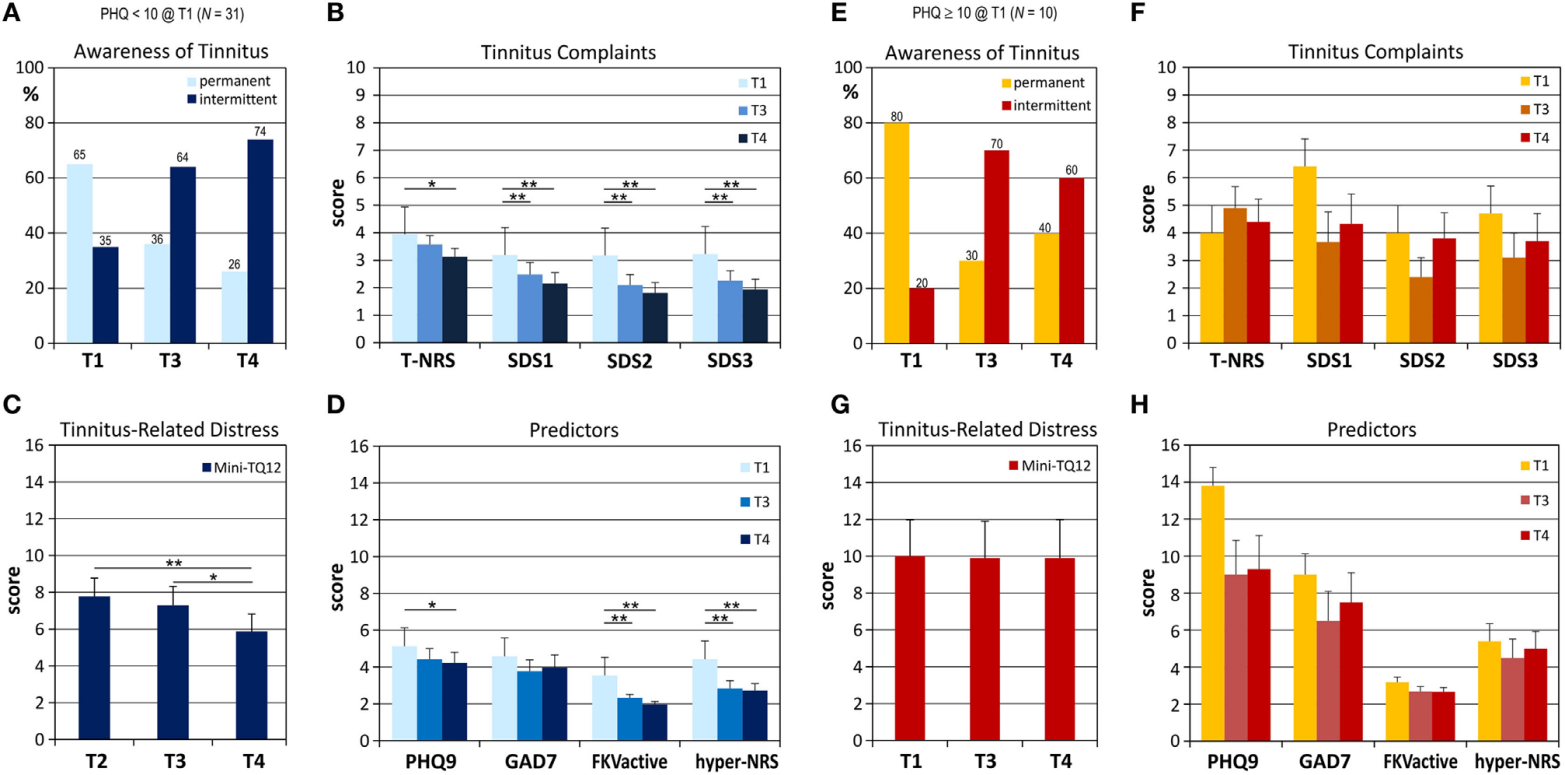
Development of tinnitus complaints and variables that were identified as predictors for future tinnitus intensity for the group with low to moderate levels of depression [**(A–D)**, blue columns], and the group with high levels of depression, [**(E–H)**, orange-red bars]. Means and respective SEs are shown. **(A,E)** Awareness of the tinnitus, **(B,F)** perceived tinnitus loudness (T-NRS), and distress as assessed by the Sheehan scales (SDS), and **(C,G)** with the Mini-TQ12. **(D,H)** Predictor variables are levels of depression (PHQ9) and anxiety (GAD7), active coping with illness (FKVactive) and oversensitivity to sound (hyper-NRS). Values for all tinnitus measures dropped in the group with low PHQ9 scores at T1 with all reductions reaching statistical significance, while there was no significant decrease for these factors in the group with high depression levels. Also, scores of all predictor variables dropped significantly in the former while any reduction observed in the latter group did not reach statistical significance between assessments. *p < 0.05; **p < 0.01.

For 73% with persisting tinnitus, the tinnitus had started suddenly whereas it began gradually in the remaining 27%, and 68% reported permanent tinnitus (Figure 2). Tinnitus onset coincided with notable HL in 17%, 26 localized tinnitus to either left (16) or right ear (10), whereas 15 heard it on both ears (10) or within the head (5). The tinnitus sound was characterized as whistling (49%), hissing (10%), complex (34%), or other (7%). Between onset and T1, tinnitus loudness had augmented in 17% and lessened in 10%, while tinnitus-related distress had worsened in 27% and lessened in 7%. T-NRS at T1 was 5 or less in 76%, and SDS scores were 5 or below in all scales in 63% of the study participants, while 15% reported a score above 5 in all scales. At T2, 30% presented with a decompensated tinnitus indicated by a sum score of 13 or above in the Mini-TQ12.

#### Hearing Loss

Audiograms were available from 39 participants. In 9 thresholds did not exceed 20 dB in any of the measured frequencies, 20 had mild to moderate HL exceeding 20 dB, 9 presented with severe HL exceeding 50 dB in at least one frequency on one ear, and one presented with single sided deafness. In unilateral tinnitus, HL was more pronounced at the tinnitus ear, and HL differences were smaller for frequencies below 2 kHz but larger for frequencies above 6 kHz as compared to bilateral tinnitus (Figure 1).

### Change of Tinnitus Complaints during Study Period

Almost 90% of the study participants developed a chronic tinnitus. At each assessment, participants were asked whether their tinnitus had changed since onset, and until T4 about half experienced stable loudness and distress, while increases were reported by 7 and 15%, respectively (Table 4). Localization and sound quality remained essentially constant, but time of awareness lessened considerably from 68% with permanent tinnitus at T1 to 29% at T4.

**Table 4:** Response to question whether tinnitus had changed since onset.

Change since onset					
		T1	T2	T3	T4
Perceived Loudness	None	28 (68.3%)	17 (41.5%)	20 (48.8%)	20 (48.8%)
	More	7 (17.1%)	3 (7.3%)	3 (7.3%)	3 (7.3%)
	Less	4 (9.8%)	19 (46.3%)	18 (43.9%)	18 (43.9%)
	No statement	2 (4.9%)	2 (4.9%)	–	–

Perceived distress	None	19 (46.3%)	15 (36.6%)	23 (56.1%)	20 (48.8%)
	More	11 (26.8%)	4 (9.8%)	4 (9.8%)	6 (14.6%)
	Less	3 (7.3%)	19 (46.3%)	11 (26.8%)	15 (36.6%)
	No statement	8 (19.5%)	3 (7.3%)	3 (7.3%)	–

Change in hearing if tinnitus onset was associated with hearing impairment	None		21 (51.2%)	12 (29.3%)	10 (24.4%)
	Better		7 (17.1%)	4 (9.8%)	2 (4.9%)
	Worse		2 (4.9%)	–	1 (2.4%)
	No statement		11 (26.8%)	25 (61%)	28 (68.3%)

In contrast, average T-NRS did not change significantly during the study period (mean ± SD: T1: 3.95 ± 1.66/T4: 3.44 ± 1.98), and therefore, initial reports at least of those who retain their tinnitus are likely to reflect perceived loudness in the chronic state. There exists no estimate for a significant clinical change for the NRS-loudness estimate, but one can assume that a change of 3 points or 27% on the 0–10 NRS represents a noticeable change. Applying this criterion, two participants experienced significant worsening of tinnitus loudness between T1 and T4, while it lessened significantly in 6 and remained stable in a majority of 80.5%. In contrast, subjective reports (Table 4) indicated 3/18 with noticeably increased/decreased loudness. Thus 12 participants thought that their tinnitus loudness had decreased since tinnitus onset despite indicating a reduction of less than 3 on the T-NRS scale, and one experienced an increase in tinnitus loudness despite a difference of less than 3 points on the T-NRS scale. Therefore, a change of 3 points appears to be a rather conservative measure for a relevant change of tinnitus loudness.

Reduction of tinnitus-related distress was statistically significant with both assessment instruments, the SDS and the Mini-TQ12. Between T1 and T4, SDS1 reduced from 3.82 ± 2.43 to 2.69 ± 2.56 (χ^2^ = 11.642; *p* = 0.003**); SDS2 reduced from 3.38 ± 2.15 to 2.29 ± 2.45 (χ^2^ = 16.487; *p* = 0.004**), and SDS3 reduced from 3.60 ± 2.23 to 2.37 ± 2.46 (χ^2^ = 13.795; *p* = 0.001**). Average scores in the Mini-TQ12 were 8.32 ± 5.76 at T2 and 6.80 ± 5.65 at T4 (*F* = 4.425; *p* = 0.015*). *Posthoc* tests with Bonferroni correction revealed that for the 3 SDS scales reductions between T1 and T3 and between T1 and T4 were highly significant, and for the Mini-TQ12 a significant decrease was present between T2 and T4. Average reductions between first and last assessment were small, however (SDS1: 1.2 ± 1.92; SDS2: 1.05 ± 2.10; SDS3: 1.20 ± 2.09, Mini-TQ12: 1.63 ± 3.55). As for the T-NRS scale, a reduction of 3 points in the SDS scales may be seen as a clinically relevant difference. According to this criterion, tinnitus complaints remained stable in 80% (SDS1), 73% (SDS2), and 75% (SDS3), while reduction of complaints between T1 and T4 was reported by 20%/25%/23% and worsening was reported by one individual for each of the SDS2 and SDS3 scales. According to the literature, a reduction of at least 5 points or 21% on the 0–24 point Mini-TQ12 may be seen as a criterion for a relevant decrease on this 24 point scale (20, 39). According to this criterion, the Mini-TQ12 score worsened significantly in 10% and ameliorated in 20%, while responses to the question whether tinnitus-related distress had changed since onset were improvement for 37%, and worsening for 15% (Table 4).

All instruments indicate, that tinnitus-related distress is stable for the majority within a short time interval following onset, and although average reductions were statistically significant, clinically relevant changes were seen only in few individuals. Regarding a detection of relevant changes, assessments with the SDS scales were the most conservative measure. Also, a 5 point increase in the Mini-TQ12 appears to be a rather conservative criterion for a relevant change, but it constituted a more sensitive instrument for the detection of change in tinnitus-related distress than the SDS scales, and measures derived from this questionnaire can be compared across studies.

### Predictors of Chronic Tinnitus Severity

In order to find potential predictors for chronic tinnitus-related distress at T4, bivariate correlations were calculated for all predictor variables at T1 with the outcome variables at T4. For unilateral tinnitus, HL at 2 kHz on the tinnitus ear showed a strong correlation with T-NRS at T4 (*r* = 0.626; *p* = 0.001), while this was not evident for the whole sample if HL was averaged across ears for those with bilateral tinnitus or tinnitus localized to the head. Bivariate correlations did not reach the Bonferroni-corrected *p*-level for significance (*p* = 0.0009) except for the correlations between the SDS scales at T1 and T4, and for the Mini-TQ12 at T2 and T4, (Table 5). These findings indicated that tinnitus complaints shortly after onset are potential predictors for the severity of chronic complaints. Furthermore, PHQ9, GAD7, hyper-NRS, FKVactive, and FKVdistraction at T1 exhibited correlation levels with outcome variables at T4 (Table 5) that were strong enough to be considered for the regression analysis.

**Table 5:** Pearson correlation coefficients (*r*) for bivariate correlations between predictor variables at T1 and outcome variables at T4 are shown together with *p*-values.

Predictors	Outcomes				
	T4-T-NRS	T4-SDS1	T4-SDS2	T4-SDS3	T4-Mini-TQ12
**Same variable at T1 or T2, respectively**					
*r*	**0.488**	**0.730**	**0.595**	**0.610**	**0.807**
*p*	**0.001**	<0.0001*	<0.0001*	<0.0001*	<0.0001*

**T1 hyper-NRS**					
*r*	0.252	**0.425**	**0.324**	**0.331**	**0.355**
*p*	0.112	**0.010**	**0.039**	**0.035**	**0.023**

**T1 PHQ9**					
*r*	**0.342**	**0.396**	**0.371**	**0.347**	**0.459**
*p*	**0.029**	**0.017**	**0.017**	**0.026**	**0.003**

**T1 GAD7**					
*r*	**0.366**	**0.352**	**0.366**	**0.360**	**0.466**
*p*	**0.019**	**0.035**	**0.019**	**0.021**	**0.002**

**T1 FKVdepressive**					
*r*	0.051	0.047	−0.18	0.044	0.221
*p*	0.757	0.787	0.913	0.786	0.170

**T1 FKVactive**					
*r*	**0.424**	**0.397**	**0.407**	**0.424**	**0.378**
*p*	**0.006**	**0.018**	0.009*	**0.006**	**0.016**

**T1 FKVdistraction**					
*r*	**0.390**	**0.381**	0.306	**0.321**	0.231
*p*	**0.013**	**0.024**	0.055	**0.043**	0.152

**T1 FKV search for meaning**					
*r*	0.104	0.177	−0.030	0.041	0.066
*p*	0.521	0.309	0.852	0.801	0.687

**T1 FKV trivialization**					
*r*	0.203	0.074	0.037	0.043	0.212
*p*	0.208	0.673	0.821	0.793	0.189

As this was an exploratory study, variables with a *p*-value below 0.05 and a correlation coefficient *r* above 0.300 (bold letters) were included in the step-wise regression analysis for the respective outcome variable. The Bonferroni-corrected *p*-value indicating statistical significance was *p = 0.0009.

Separate stepwise regression analyses for each of the outcome variables and the predictors with sufficiently strong correlations showed that the level of complaints at T1 in each scale was a good predictor for scores in that scale at T4. Mini-TQ12 scores at T4 were explained to 70% by the factors Mini-TQ12 at T2 and PHQ9 at T1. For Tinnitus loudness, the factors T-NRS, FKVactive, and PHQ9 all at T1 explained 50% of the variance. Scores in the SDS1 scale at T4 were explained to 53% the SDS1 score at T1, whereas the SDS2 score at T4 was explained by 35% by the SDS2 score at T1, and the SDS3 score at T4 was explained by 49% by the SDS3 score and the FKVactive score at T1 (Table 6). Thus, tinnitus-related distress can be foreseen in the acute state, and best predictors were the Mini-TQ12 and the PHQ9.

**Table 6:** Results of step-wise regression analysis.

Predictor at T1	Outcome at T4				
	T-NRS	SDS1	SDS2	SDS3	Mini-TQ12
Explained variance	50%	53%	35%	49%	70%
*R*^2^/adj *R*^2^	0.497/0.455	0.527/0.509	0.354/0.337	0.485/0.457	0.695/0.678
*F*	11.841	30.026	20.819	21.483	42.111

**Same variable at T1/T2**					
Stand. beta_in_	0.444	0.726	0.595	0.540	0.734
*p*	0.001**	<0.001**	<0.001**	<0.001**	<0.001**

**T1 PHQ9**					
Stand. beta_in_	0.283				0.218
*p*	0.024*				0.030*

**T1 FKVactive**					
Stand. beta_in_	0.376			0.350	
*p*	0.003**			0.007**	

Stepwise linear regression analysis was performed separately for each of the outcome variables. Variables included in the analysis were those with sufficient bivariate correlations of r > 0.300 and a p < 0.05 with the respective outcome in the bivariate analyses as shown in Table 5. For the regression analysis significant predictors (*p < 0.05; **p < 0.01) together with the standardized beta values are shown for each of the outcome variables.

### Groups with High versus Low Depression Scores

One of our initial hypotheses was that a high level of depression at tinnitus onset promotes development of a decompensated chronic tinnitus. We therefore compared the group with low level of depression, i.e., a PHQ9 score below 10 at T1 (*N* = 31) and the group with conspicuous levels of depression at T1 (*N* = 10), and found that a high level of depression around tinnitus onset allows a distinction between problematic and unproblematic tinnitus at T4. Tinnitus symptoms were lower and reduced significantly during the study interval in the group with low depression scores at T1, while they remained unchanged for the group with PHQ9 scores of 10 or above (Figure 2). In addition, GAD7 scores and hypersensitivity to external sounds was more pronounced in the latter group, and members of the group with high depression scores were more likely to undergo a tinnitus-specific therapy during the study interval (70%) compared to the group with an inconspicuous PHQ9 score (58%).

## Discussion

Aim of the present study was to describe the transition from acute to chronic tinnitus, identify factors associated with remission of the tinnitus and to identify predictors for the development of chronic disabling tinnitus. To our knowledge, this is the first study that aims to investigate individuals with a first episode of tinnitus who seek medical help from private practices of otolaryngologists. Former studies investigated tinnitus sufferers that were hospitalized for the treatment of sudden HL (6, 7), took part in a psychological therapy for acute tinnitus (14), or army personal developing tinnitus shortly after exposure to high noise levels (9).

### Study Sample

The characteristics of our study sample suggest that patients seeking help from otolaryngologists for acute tinnitus are not necessarily the ones with high tinnitus-related distress or loud tinnitus as indicated by the rather moderate averages of these measures in comparison to those assessed with the same instruments in a large number of subjects with chronic tinnitus (4: T-NRS: 6.0 ± 2.5; Mini-TQ12: 10.4 ± 6.4). Averages are comparable, however, to those found by earlier studies with acute tinnitus patients (5, 6, 17). The authors of one study (17) assume that tinnitus questionnaires may underestimate tinnitus-related distress in acute patients because they were developed for the chronic condition. Alternatives which are not mutually exclusive are that patients with high tinnitus-related distress consult other specialists, or that tinnitus-related distress increases over time. Another observation was that not many patients consult an otolaryngologist early after tinnitus onset. This is in line with a former report, stating that the majority seeks medical help only years after tinnitus onset [(40); Lockwood et al. (41)], and with the results of a recent review on the literature (12). Of note is also that average scores of depression and anxiety are similar to those found with the same instruments for a large sample with chronic tinnitus [(4): PHQ9: 7.1 ± 5.5; GAD7: 6.0 ± 4.8] which is considerably higher than in the German normative samples [(32): PHQ9: 2.9 ± 3.5; 29: GAD7: 3.0 ± 3.4].

In the present sample, average age is 41.4 ± 15.6 years in the group with persisting tinnitus and 41.0 ± 14.8 years in the group with remitting tinnitus. This is lower than in a sample with tinnitus following sudden HL [(6): 47.3 years, range 19–78], but comparable to the average of a large group with chronic tinnitus who retrospectively reported age at tinnitus onset to be 42.4 ± 13.5 years (42). In that report, study participants with later tinnitus onset experienced a more distressing tinnitus right from the beginning (42). Whereas in a preliminary analysis of the present study with the data of 28 participants (20), we also found a significant correlation of moderate strength between age at tinnitus onset and tinnitus-related distress, we could not confirm this finding for the Mini-TQ12, the instrument used by Schlee et al. (42), in this larger sample, but found a stronger correlation between age at onset and tinnitus-related distress as assessed by the SDS2 and SDS3 scales at T4. In addition, we found strong correlations between age at onset and HL at frequencies of 3 kHz and above (*r*: 0.384–0.718). While prevalence of HL increases with age, it may go undetected by the affected individuals (43) and it is not known whether the sample investigated by Schlee et al. (42) had hearing levels within the normal range at tinnitus onset. HL increases the risk for developing tinnitus, and tinnitus-related distress may be augmented by the distress the participant experiences because of the accompanying HL (42).

### Tinnitus Onset, Remission, and Transition to Chronic Condition

Remission rate was about 11% in the current study, it occurred more often in individuals who developed tinnitus concomitant with a noticeable HL, and remission usually occurred during the first weeks after onset, resembling the time course of recovery of hearing after incidences of acute HL (7, 11). The majority of our sample did not experience worsening of hearing concomitant with tinnitus onset, but this percentage is higher in the group with complete remission. Higher remission rates are commonly reported in studies with tinnitus in response to acute auditory insults (9), likely because of recovery after acute acoustic insult (11). Furthermore, hearing recovery appears to be age- and frequency dependent, as it is more complete in younger individuals and at frequencies below and above 4 kHz (44). As acute temporary threshold shifts are known to recover to some extent resulting in a less severe permanent threshold shift (11), tinnitus remission is more likely if its appearance is associated with acute worsening of hearing level. In animal models, threshold shifts of up to about 50 dB immediately after a single-noise exposure may recover completely, and recovery has been reported for periods extending up to 3 weeks. But even with complete recovery of hearing threshold large numbers of synapses between hair cells and primary afferent neurons may be lost resulting in hearing deficits that are not necessarily detected by audiometry (11).

Recovery in hearing seems to precede decreases in tinnitus loudness or tinnitus remission (45), and complete remission sets in earlier, i.e., by day 7, and benefits a significantly higher share of patients with mild-moderate HL as compared to those with more profound HL in whom remission was observed in a period of up to 30 days. Also, hearing impairment and tinnitus in response to an ear infection tend to recover completely (46). Interestingly, the ones with remitting tinnitus in our sample indicated at T1 that loudness or annoyance of their tinnitus had decreased since onset suggesting a gradual recovery of hearing thresholds and reduction of tinnitus severity soon after tinnitus onset. Mühlmeier et al. (7) report that tinnitus remission lagged complete hearing recovery, and that complete hearing recovery and complete tinnitus remission were not independent of each other confirming the association between both factors. Taken together, data suggest that remission can be expected with substantial recovery of hearing threshold, for instance after incidences of sudden HL or acoustic trauma while remission is not to be expected if tinnitus onset was not associated with a recent incidence of HL.

Most pronounced was the reduction of tinnitus awareness between T1 and T3. It did not necessarily result in a less distressing tinnitus, however. 59% retained the level of tinnitus-related distress experienced at T2 until T4, while some habituated as indicated by lower distress 6 months after onset. About 10% reported significant worsening of tinnitus-related distress during the study. Hence, tinnitus-related distress reported in the acute condition is likely to be representative for the chronic condition. This finding is corroborated by the results of the regression analysis. Similarly, perceived loudness of the tinnitus was stable between T1 and T4 in most of the study participants, which is no surprise since for most tinnitus onset was not related to worsening of the hearing. Taken together, tinnitus remission occurred early, but the tinnitus remained in 90%, and tinnitus complaints did not change in the majority. Therefore, initial complaints about high tinnitus-related distress and perceived loudness require immediate therapeutic action.

The time course of transition from the acute to the chronic condition is not entirely clear. Whereas, some define duration of up to 3 months as subacute and a tinnitus lasting at least 6 months as being chronic (12), others assume the chronic condition is reached already after 3 months (19). Assessments in the present study were chosen to investigate these time intervals and together with studies on spontaneous remission after sudden HL (7) indicate that chronification of the tinnitus is taking place during the first days or weeks after onset. This is nicely shown by an interventional study (18) reporting that adults with unilateral tinnitus who are treated with intratympanic dexamethasone or saline both together with oral gingko biloba, the latter thought to be a placebo treatment, reduced awareness of the tinnitus within a month of tinnitus onset, and also led to reductions of tinnitus loudness and annoyance within the following 4 weeks irrespective of the treatment. In contrast, Holgers et al. (8) report that between the first consultation which took place during the first 6 months after tinnitus onset and the endpoint of the study one year later, tinnitus symptoms decreased in 75% of the study participants, whereas the rest had symptoms that did not decrease over time.

### Hearing Loss As a Predictive Factor

Hearing loss has been identified as the most relevant etiologic factor and as the probable trigger for tinnitus, although non-auditory factors may ameliorate or worsen the tinnitus (47). In the present sample 25% of those with unilateral tinnitus and 20% with bilateral tinnitus did not have an overt HL in the standard audiogram indicated by thresholds not exceeding 20 dB at any frequency. For those with unilateral tinnitus, thresholds in the affected ear were worse for the tinnitus ear which is in line with findings for patients with idiopathic sensorineural HL (48). Noteworthy, in our sample is the high correlation between HL at 2 kHz and future tinnitus loudness which has not been described before. In accordance, Mühlmeier et al. (7) report that tinnitus loudness 3 months after tinnitus onset was statistically significantly correlated with the level of PTA both at baseline and at 3 months.

Reasons why a tinnitus was heard despite normal hearing thresholds might be due to HL in frequencies above 8 kHz as seen in a recent study in patients suffering from acute tinnitus (49). Furthermore, tinnitus in normal hearing subjects may be due to cochlear pathologies undetected by the audiogram (11), or to a less well functioning efferent auditory system (50).

### Depression and Anxiety As Predictive Factors

Variables with notable correlations with future tinnitus severity were the levels of depression and anxiety at T1. In the group with high levels of depression at T1, tinnitus loudness and distress did not diminish with time, but even augmented in most of those fulfilling the criterion for Major Depression. High levels of depression have been associated with chronic disabling tinnitus (4). Holgers et al. (8) report that above all symptoms of depression and anxiety together with HL and physical immobility are predictors for severity of the tinnitus, and high depression levels were not observed in any one of their patients that showed tinnitus remission.

In our sample, we find significant reductions of tinnitus-related distress, loudness, and the level of depressive symptoms only in the group with low levels of depression at T1, while neither factor reduced during the study interval if PHQ9 scores at T1 indicated conspicuous problems with depression. In addition, we found a significant correlation between tinnitus-related distress at onset and chronic tinnitus-related distress. This supports the notion, that high levels of tinnitus-related distress in particular in combination with high levels of depressive symptoms are indicators for the potential development of incapacitating tinnitus. Therefore, it is indicated to assess tinnitus-related distress in acute tinnitus patients, and this should always be accompanied by screening of the level of depression. As an additional measure, the level of anxiety could be assessed, as levels of depression and anxiety show strong correlations and it was found that during transition, for instance in the course of chronification, levels of anxiety show a stronger correlation with tinnitus symptoms than indicators of depression [(8, 17, 51)—this Frontiers Topic].

Data indicate the importance of interventions already during the acute phase of tinnitus, as previously suggested (14). Effects of such interventions should be more pronounced in acute tinnitus associated with high levels of depression. Therefore, we consider early psychological/psychotherapeutic intervention to be important to prevent the development of a decompensated chronic tinnitus that severely impairs life quality of affected individuals.

### Active Coping with Tinnitus Is Maladaptive

Results of the regression analysis indicate that active coping is maladaptive in regard to tinnitus. Active coping with the tinnitus reduces significantly in the group with an inconspicuous level of depression at T1, while no reduction is found in the group with high levels of depression. It therefore appears that those with psychological problems tend to adhere to this strategy.

### Limitations

Some limitations of the study should be named. As in the other few longitudinal studies, the number of participants is low. Furthermore, because measures on tinnitus-loudness and distress are not available on a large-scale for the acute condition, it cannot be decided, whether our sample is representative for the acute tinnitus population, but these measures may rather serve as a start to describe acute tinnitus patients.

Only few individuals appear to consult an otolaryngologist shortly after noticing the appearance of a tinnitus (40), and nowadays acute tinnitus patients may better be contacted via internet sources as suggested by a recent publication [(52), this Frontiers Topic]. An alternative which is not mutually exclusive is that perceived tinnitus loudness and tinnitus-related distress are mild at first but may increase over the life span. An increase of tinnitus loudness is likely because with advancing age most suffer from presbyacusis, and it has been shown that tinnitus loudness rather increases with age. An increase of tinnitus-related distress is not self-explicable as many of those with long-existing tinnitus report adjustment to the tinnitus and experience a decrease of tinnitus-related distress (4). It may be possible, however, that tinnitus-related distress increases for instance in individuals with high levels of depression or anxiety.

Because perceived tinnitus loudness and tinnitus-related distress were both low to moderate at T1/T2 reductions of tinnitus loudness and distress may not have been detected. We used the GAD7 for screening anxiety as it is a short freely available and validated instrument for screening GAD and was reported to perform almost as well for detecting other types of common anxiety disorders (29). A very recent publication of the same group (53) suggests that the first four items discriminate better than the last three items with respect to latent anxiety, and therefore suggest to use a 2item GAD version (GAD2) which will be even more time-economic. Nonetheless, we decided to report the sum scores of the GAD7 as they have been reported for a normative sample (29) and in former tinnitus studies (4, 33) but suggest to monitor the literature regarding further developments.

## Conclusion

(1) Patients that present with acute tinnitus at otolaryngology practices are not a homogenous group in which psychological aspects are important to predict future progression of the tinnitus. Therefore, mental health should be assessed early on, for instance with the readily available PHQ9 and GAD7 questionnaires used in this study.
(2) If levels of depression or anxiety are elevated, patients should be referred to specialists treating these conditions.
(3) Results support early manifestation of tinnitus-related distress, and that development of disabling tinnitus is fostered by poor mental health.

Therefore, we suggest to screen depression symptoms and tinnitus-related distress of all tinnitus patients presenting with acute or subacute tinnitus, and to refer them to specialist evaluation and therapy if indicated. Furthermore, we suggest to consider provision with hearing prostheses, although it has to be assured first that a potentially existing oversensitivity to sounds, has reversed when using hearing aids.

## Ethics Statement

Study design and procedures were approved by the Ethics Committee II of Heidelberg University at the Medical Faculty Mannheim (2013-541N-MA).

## Author Contributions

EW-F designed the study, collected part of the data, analyzed the data, and wrote the manuscript. RD interpretation of data, AG collected and analyzed part of the data, WD designed the study, JS critical review, KH critical review, IR recruitment and interpretation of data.

## Conflict of Interest Statement

The authors declare that the research was conducted in the absence of any commercial or financial relationships that could be construed as a potential conflict of interest. The reviewer SW and handling Editor declared their shared affiliation.
